# Summer Resource Selection and Identification of Important Habitat Prior to Industrial Development for the Teshekpuk Caribou Herd in Northern Alaska

**DOI:** 10.1371/journal.pone.0048697

**Published:** 2012-11-05

**Authors:** Ryan R. Wilson, Alexander K. Prichard, Lincoln S. Parrett, Brian T. Person, Geoffry M. Carroll, Melanie A. Smith, Caryn L. Rea, David A. Yokel

**Affiliations:** 1 The Wilderness Society, Anchorage, Alaska, United States of America; 2 ABR, Inc. – Environmental Research & Services, Fairbanks, Alaska, United States of America; 3 Alaska Department of Fish and Game, Fairbanks, Alaska, United States of America; 4 North Slope Borough Department of Wildlife Management, Barrow, Alaska, United States of America; 5 Alaska Department of Fish and Game, Barrow, Alaska, United States of America; 6 Audubon Alaska, Anchorage, Alaska, United States of America; 7 ConocoPhillips Alaska, Inc., Anchorage, Alaska, United States of America; 8 United States Bureau of Land Management, Fairbanks, Alaska, United States of America; University of California, Berkeley, United States of America

## Abstract

Many caribou (*Rangifer tarandus*) populations are declining worldwide in part due to disturbance from human development. Prior to human development, important areas of habitat should be identified to help managers minimize adverse effects. Resource selection functions can help identify these areas by providing a link between space use and landscape attributes. We estimated resource selection during five summer periods at two spatial scales for the Teshekpuk Caribou Herd in northern Alaska prior to industrial development to identify areas of high predicted use for the herd. Additionally, given the strong influence parturition and insect harassment have on space use, we determined how selection differed between parturient and non-parturient females, and between periods with and without insect harassment. We used location data acquired between 2004–2010 for 41 female caribou to estimate resource selection functions. Patterns of selection varied through summer but caribou consistently avoided patches of flooded vegetation and selected areas with a high density of sedge-grass meadow. Predicted use by parturient females during calving was almost entirely restricted to the area surrounding Teshekpuk Lake presumably due to high concentration of sedge-grass meadows, whereas selection for this area by non-parturient females was less strong. When insect harassment was low, caribou primarily selected the areas around Teshekpuk Lake but when it was high, caribou used areas having climates where insect abundance would be lower (i.e., coastal margins, gravel bars). Areas with a high probability of use were predominately restricted to the area surrounding Teshekpuk Lake except during late summer when high use areas were less aggregated because of more general patterns of resource selection. Planning is currently underway for establishing where oil and gas development can occur in the herd’s range, so our results provide land managers with information that can help predict and minimize impacts of development on the herd.

## Introduction

Across the circumpolar north, many caribou and reindeer (*Rangifer tarandus*) populations are exhibiting synchronous declines [1]. While numerous hypotheses have been proposed for these declines (e.g., climate change; [1]), habitat loss due to human development has received the most attention. In southern Canada, for example, the predominant cause of woodland caribou population declines is thought to be habitat alteration due to industrial activities [2]. Loss of habitat from human development can result directly from the conversion of habitat to infrastructure or indirectly through avoidance behavior of caribou to the presence of activity associated with infrastructure [3]–[5]. For example, even though <1% of the landscape in a study from Norway was directly modified by human development, 75% was within 4 km of development; the distance reindeer generally avoided development [3]. Similarly, in Canada, Dyer et al. [6] found that up to 48% of their study area could receive reduced use by caribou due to disturbance from human development. Given these realities, there is concern that the cumulative effects of increased industrial development in the arctic will have negative population-levels effects on many caribou herds [2].

Because even small development footprints can lead to significant habitat loss [3], it is important to identify where high value habitat occurs for caribou before development proceeds into a herd’s range. A recent study of two herds in Québec, Canada, however, highlighted the drawback of relying solely on range estimates for identifying these areas [7]. In their study, Taillon et al. [7] demonstrated that using only three years of data to derive range estimates for the establishment of long-term conservation areas, resulted in <20% protection of each herd’s calving ground, annually. Given that we will rarely know how many years of data are needed to capture the range of annual variability in range use, and are unlikely to have the benefit of multiple years prior to expanded development, we suggest a more mechanistic approach to identifying high value wildlife habitat.

Estimating resource selection patterns for a herd can reveal what habitat features are selected (or avoided) on the landscape. Resource selection functions (RSFs; [8]) are a valuable tool for identifying important areas for a population so that the quantity and distribution of those areas across a population’s range can be assessed [9]. Results from RSFs can then be used to help predict and minimize negative impacts to caribou habitat from development, especially during periods of enhanced sensitivity to human activity associated with development (i.e., calving; [4]). It is important, however, that RSFs be estimated before development begins because patterns of resource selection can be altered in the presence of infrastructure [10]. Additionally, estimates obtained prior to development can be used to assess if or how patterns change once development exists.

**Figure 1:**
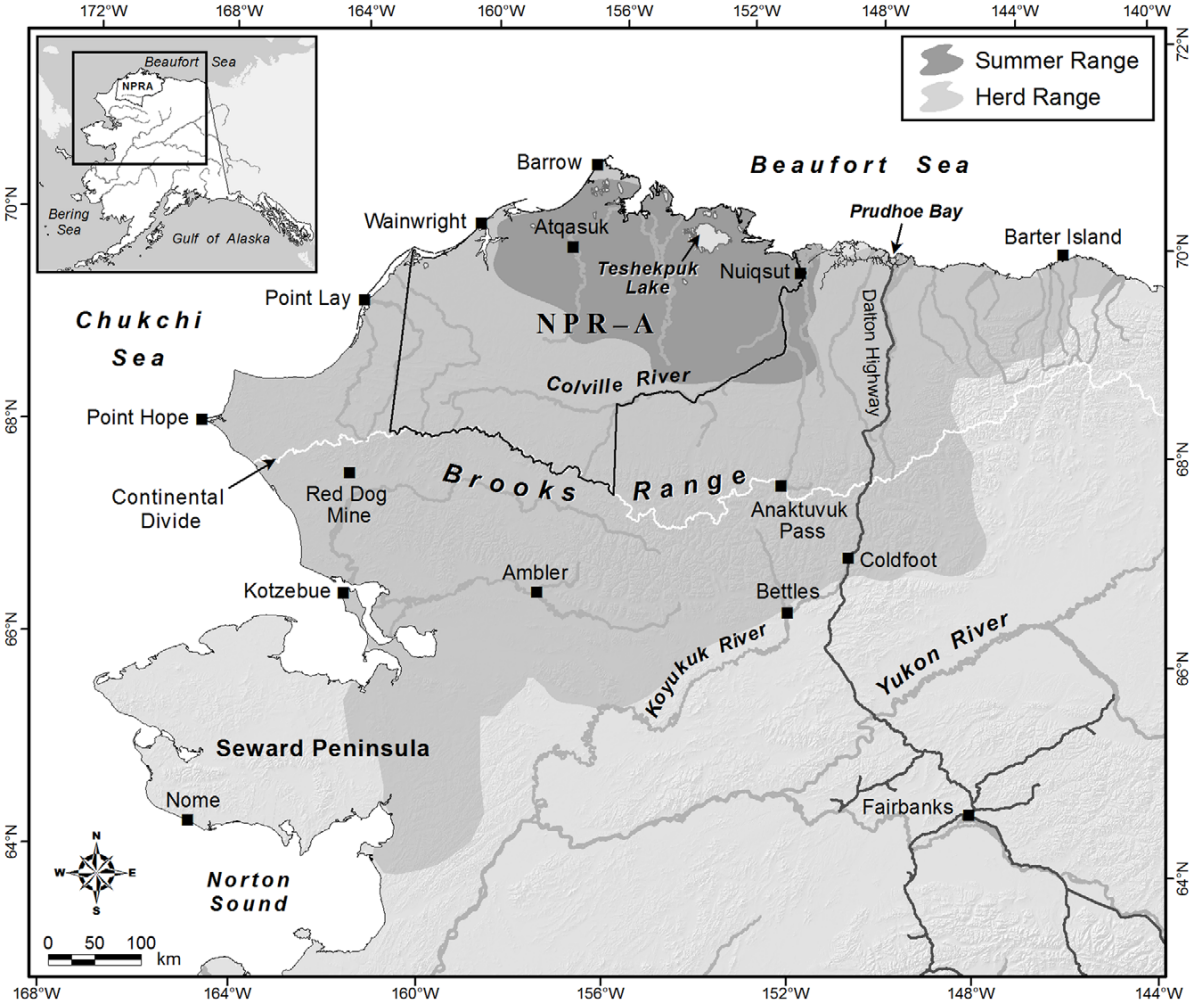
Teshekpuk Caribou Herd summer and annual ranges in relation to the National Petroleum Reserve Alaska.

The Teshekpuk Caribou Herd resides in northern Alaska with nearly all of its annual range overlapping with the National Petroleum Reserve-Alaska (NPRA; Fig. 1). Limited industrial development has occurred inside NPRA, but planning efforts are currently underway to determine how and where oil and gas development will be allowed to proceed in the coming decades. Research has been conducted to document the herd’s seasonal ranges over the last two decades [11], but as stated above, this might be insufficient for identifying high value areas for the herd prior to development. The importance of identifying high value areas for the herd during summer are highlighted by the adjacent Central Arctic Herd which has been exposed to oil development for the past 30 years on their summer range. After development occurred in a portion of the core calving area, the calving area shifted to the south [12], vegetation composition of the new areas differed [13], and displaced females showed evidence of lower productivity and calf survival [4]. Additionally, increased insect harassment associated with warm weather can impair summer weight gain by caribou [14] leading to increased winter mortality [15]–[16]. Shifts in space use to reduce insect harassment may be an important mechanism for reducing lost foraging opportunities due to harassment, but movements to these areas could be impaired by infrastructure.

By estimating resource selection functions at multiple spatial scales through summer we can also better understand the choices caribou make in selecting their seasonal ranges. Considering multiple scales of selection is particularly important for migratory species, such as caribou, where individuals may select certain features of the landscape for placement of seasonal ranges (i.e., landscape scale), but show different patterns of selection within seasonal ranges as they search for forage (i.e., patch scale; [17]). For example, migratory elk (*Cervus elaphus*) select areas with low predator density when choosing seasonal ranges, but select for forage quality at finer spatial scales [9]. Studying broader scale selection, such as at the landscape scale (i.e., 2nd order selection; [18]), can help identify where seasonal ranges might be supported on the landscape [19]. Conversely, patch-scale analysis can be informative for understanding what features individuals prefer to carry out specific behaviors (e.g., foraging), or how they respond to infrastructure [20].

The goal of this study was to estimate changes in resource selection across five biologically-relevant periods of summer at both the landscape and patch scales to better understand where high value habitat is distributed across the herd’s summer range prior to industrial development. Unlike other ungulate populations which migrate primarily between winter and summer ranges, caribou in the Arctic require further seasonal movements within the summer range to track quickly-changing vegetation conditions and avoiding the large energetic costs of insect harassment. Thus, we also determined how selection differed between parturient and non-parturient females, and between periods with and without insect harassment. By estimating RSFs, we provide land managers with information on where development of the NPRA could occur to lower the potential impact on the TCH.

## Methods

### Ethics Statement

This research was approved by the State of Alaska, Department of Fish and Game, Animal Care and Use Committee (#07–13).

**Table 1:** Description and area of vegetation types across the National Petroleum Reserve-Alaska.

Vegetation Type	Area (km^2^)	Area (%)	Description
Flooded	6549	7	Continuously flooded areas composed of 25–50% water, with the dominant plant being *Carex aquatilis*
*Carex aquatilis*	1628	2	Shorelines of lakes or ponds dominated by *Carex aquatilis*
Riverine	4689	5	Areas associated streams, rivers, or coastal beaches with sparse vegetation (<10% cover); a combination of dunes, dry sand, sparsely vegetated, and barren ground.
Wet Tundra	4646	5	Areas of super-saturated soil or standing water composed of 10–25% water. The dominant plant species is *Carex aquatilis*
Sedge-Grass Meadow	5744	6	Continuous mats of grass and sedges composed of *Carex aquatilis*, *Eriophorum angustifolium, E. russeolum, Arctagrostis latifolia,* and *Poa arctica.*
Tussock Tundra	25857	27	Areas of well-drained soils containing tussocks formed predominantly by *Eriphorum vaginatum*
Moss-Lichen	962	1	Low-lying lakeshores and dry sandy ridges dominated by moss and lichens
Dwarf Shrub	41037	43	Ridges and well-drained soils dominated by shrubs, primarily *Salix* spp., *Betula nana*, or *Ledum palustre*, <30 cm in height; frequently occurs over a substrate of tussocks.
Low Shrub	4246	4	Areas along small streams and rivers, or along hill sides, dominated by shrubs primarily *Salix* spp., *Betula nana*, *Alnus crispa*, or *Ledum palustre*, 0.3–1.5 m in height.

### Study Area

Our study area was restricted to the summer range of the TCH which lies almost entirely within the NPRA (Fig. 1). The TCH summer range occurs on the central coastal plain in northern Alaska (Fig. 1). The area is characterized by low topographic relief (<60 m elevation; [11]) and a high density of lakes ranging in size from <1 km^2^ to >800 km^2^ [21]. The dominant vegetation communities within the TCH summer range are wet and moist sedge, composed mainly of cotton grass (*Eriophorum* spp.) and *Carex aquatilis*. The study area has a short snow-free growing period that lasts from June through September.

The TCH typically calves near Teshekpuk Lake, uses the area between Teshekpuk Lake and the Beaufort Sea as mosquito-relief habitat, then disperses further inland during late summer [11]. The size of the TCH is estimated at 55,000 and currently supports a large subsistence harvest and smaller non-local harvest (4,000–5,000 annually; L. Parrett, unpublished data). Wolves (*Canis lupus*), brown bears (*Ursus arctos*), and Golden Eagles (*Aquila chrysaetos*) prey on the TCH, but are relatively rare on the coastal plain [21].

**Figure 2:**
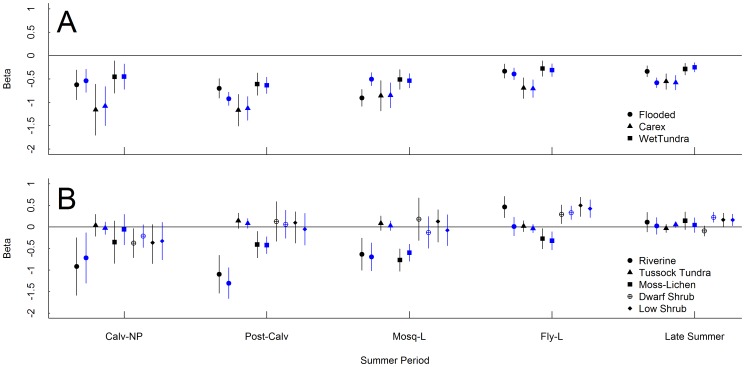
Coefficient estimates for vegetation patch types throughout summer. Results are from top resource selection models at the landscape (black) and patch (blue) scales during 5 periods of summer for the Teshekpuk Caribou Herd in Alaska, 2004–2010. These periods include calving (non-parturient females only; Calv-NP), post-calving (Post-Calv), mosquito (periods with low harassment; Mosq-L), oestrid fly (periods with low harassment; Fly-L), and late summer (Late Summer). Top panel (A) is vegetation types dominated by standing water, whereas the bottom panel (B) is all other vegetation types. Coefficient estimates with 95% CI overlapping the zero-line are considered not significantly different than selection for sedge-grass meadows (used as the reference class in all resource selection function models).

**Table 2:** K-fold cross-validation results for top resource selection function models.

	Landscape Scale		Patch Scale	
Model	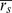	*P*-value	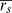	*P*-value
Calving Parturient	0.939	<0.001	0.830	0.006
Calving Non-Parturient	0.963	<0.001	0.867	0.003
Post-Calving	0.964	<0.001	0.927	0.001
High Mosquito Harassment	0.852	0.004	0.927	0.001
Low Mosquito Harassment	0.842	0.005	0.867	0.003
High Fly Harassment	0.964	<0.001	0.883	0.003
Low Fly Harassment	0.855	0.004	0.867	0.003
Late Summer	0.964	<0.001	0.648	0.049

**Table 3:** Results from top resource selection function models for post-calving and late summer at both the landscape and patch-scales for the Teshekpuk Caribou Herd in northern Alaska: 2004–2010.

	Period							
	Post Calving				Late Summer			
	Landscape Scale		Patch Scale		Landscape Scale		Patch Scale	
Coefficient	β	SE	β	SE	β	SE	β	SE
Intercept	−1.573	0.275	NA^1^	NA	0.089	0.078	NA	NA
Flooded patch	−0.700	0.107	−0.616	0.073	−0.336	0.059	−0.274	0.054
Flooded density	0.565	0.053	NA	NA	0.140	0.022	NA	NA
*Carex aquatilis* patch	−1.447	0.173	−1.117	0.130	−0.554	0.085	−0.580	0.079
*Carex aquatilis* density	0.344	0.041	NA	NA	0.540	0.020	NA	NA
Riverine patch	−1.098	0.224	−1.305	0.183	0.112	0.115	0.024	0.100
Riverine density	−0.044	0.038	NA	NA	−0.231	0.021	NA	NA
Wet tundra patch	−0.611	0.122	−0.636	0.089	−0.288	0.064	−0.252	0.051
Wet tundra density	−0.121	0.042	NA	NA	0.209	0.015	NA	NA
Sedge-grass meadow density	0.710	0.053	NA	NA	0.499	0.022	NA	NA
Tussock tundra patch	0.142	0.092	0.081	0.058	−0.033	0.049	0.054	0.035
Tussock tundra density	–2	–	NA	NA	0.461	0.018	NA	NA
Moss-lichen patch	−0.409	0.157	−0.423	0.100	0.142	0.105	0.043	0.088
Moss-lichen density	0.073	0.041	NA	NA	0.146	0.018	NA	NA
Dwarf shrub patch	0.128	0.236	0.063	0.167	−0.094	0.061	0.225	0.060
Dwarf shrub density	−6.082	0.647	NA	NA	–	–	NA	NA
Low shrub patch	0.099	0.242	−0.053	0.188	0.164	0.086	0.162	0.069
Low shrub density	−0.481	0.058	NA	NA	−0.104	0.019	NA	NA
Green-up date	0.495	0.048	NA	NA	–	–	NA	NA
Max-growth date	0.139	−0.001	NA	NA	0.057	0.017	NA	NA
Distance to coastline	0.629	0.296	–	–	0.386	0.024	–	–
Ruggedness	–	–	–	–	–	–	0.026	0.020
Ruggedness2^3^	–	–	–	–	–	–	–	–
Precipitation	0.139	0.055	NA	NA	–	–	NA	NA
Elevation	–	–	0.154	0.102	–	–	–	–

1 Indicates variable was not included in initial set of variables considered for model.

2 Indicates variable was available to be included in model, but was not included in final model.

3 Indicates a squared coefficient.

### Caribou Capture and Monitoring

Between 2004 and 2010 we captured 41 adult female caribou from the TCH with a net gun fired from a helicopter and collared them with GPS collars (Telonics, Mesa, AZ). The number of individuals monitored differed annually (i.e., 2004 = 10, 2005 = 10, 2006 = 12, 2007 = 17, 2008 = 18, 2009 = 21, and 2010 = 8), with most individuals monitored for >1 year (n = 35). We obtained locations every 3 (2004–2005) or 2 hours (2006–2010) and removed erroneous points and those with 2D fixes or a positional dilution of precision >10. These cut-offs provide the greatest improvement in mean location accuracy [22] and resulted in a 2.6% reduction in data for analysis. While this results in loss of locations for analysis, it allows for greater accuracy in the estimation of resource selection especially at fine-spatial scales [23], and there was no systematic bias with vegetation type (χ^2^ = 2.02, *P* = 0.98). We also excluded locations obtained while caribou were assumed to have joined another herd. We assumed a caribou switched herds if it was in the calving area of another herd during a subsequent calving season.

**Table 4:** Results from top resource selection function models for parturient and non-parturient females during the calving period at landscape and patch-scales for the Teshekpuk Caribou Herd in northern Alaska: 2004–2010.

	Period							
	Calving Parturient				Calving Non-Parturient			
	Landscape Scale		Patch Scale		Landscape Scale		Patch Scale	
Coefficient	β	SE	β	SE	β	SE	β	SE
Intercept	−0.028	0.366	NA^1^	NA	−0.036	0.160	NA	NA
Flooded patch	−0.182	0.194	−0.531	0.098	−0.625	0.162	−0.539	0.127
Flooded density	−0.109	0.066	NA	NA	0.139	0.058	NA	NA
*Carex aquatilis* patch	−1.032	0.288	−1.072	0.172	−1.158	0.279	−1.080	0.214
*Carex aquatilis* density	1.526	0.096	NA	NA	0.317	0.058	NA	NA
Riverine patch	−1.804	0.613	−0.813	0.333	−0.918	0.342	−0.720	0.299
Riverine density	−0.399	0.080	NA	NA	−0.263	0.054	NA	NA
Wet tundra patch	−0.122	0.215	−0.401	0.097	−0.456	0.176	−0.450	0.138
Wet tundra density	−0.257	0.063	NA	NA	−0.071	0.043	NA	NA
Sedge-grass meadow density	1.256	0.090	NA	NA	0.500	0.063	NA	NA
Tussock tundra patch	0.301	0.149	0.147	0.060	0.036	0.130	−0.029	0.075
Tussock tundra density	–2	–	NA	NA	0.198	0.052	NA	NA
Moss-lichen patch	0.344	0.352	−0.184	0.158	−0.354	0.253	−0.060	0.181
Moss-lichen density	–	–	NA	NA	–	–	NA	NA
Dwarf shrub patch	−0.803	0.240	−0.339	0.163	−0.376	0.173	−0.211	0.135
Dwarf shrub density	–	–	NA	NA	–	–	NA	NA
Low shrub patch	−0.957	0.562	−0.565	0.299	−0.365	0.248	−0.327	0.222
Low shrub density	–	–	NA	NA	–	–	NA	NA
Green-up date	0.623	0.082	NA	NA	0.582	0.262	NA	NA
Ruggedness	0.507	0.154	–	–	1.761	0.231	−0.042	0.025
Ruggedness2^3^	−0.223	0.152	–	–	−3.304	0.423	–	–

1 Indicates variable was not included in initial set of variables considered for model.

2 Indicates variable was available to be included in model, but was not included in final model.

3 Indicates a squared coefficient.

**Table 5:** Results from top resource selection function models for periods with predicted high and low mosquito and oesterid fly harassment at both the landscape and patch-scales for the Teshekpuk Caribou Herd in northern Alaska: 2004–2010.

	Period															
	Mosquito-H				Mosquito-L				Fly-H				Fly-L			
	Landscape Scale		Patch Scale		Landscape Scale		Patch Scale		Landscape Scale		Patch Scale		Landscape Scale		Patch Scale	
Coefficient	β	SE	β	SE	β	SE	β	SE	β	SE	β	SE	β	SE	β	SE
Intercept	0.573	0.175	NA^1^	NA	0.374	0.070	NA	NA	0.089	0.128	NA	NA	0.079	0.061	NA	NA
Flooded patch	−1.091	0.178	−0.924	0.103	−0.904	0.093	−0.794	0.072	−0.336	0.140	−0.506	0.096	−0.332	0.077	−0.392	0.064
Flooded density	–2	–	NA	NA	–	–	NA	NA	−0.093	0.050	NA	NA	0.032	0.026	NA	NA
*Carex aquatilis* patch	−1.169	0.288	−1.130	0.199	−1.578	0.165	−1.407	0.137	−0.858	0.207	−0.849	0.151	−0.695	0.113	−0.708	0.097
*Carex aquatilis* density	−0.112	0.059	NA	NA	0.227	0.038	NA	NA	0.365	0.048	NA	NA	0.273	0.025	NA	NA
Riverine patch	−0.476	0.346	−0.864	0.161	−0.633	0.191	−0.696	0.166	0.659	0.211	0.059	0.141	0.463	0.127	0.008	0.111
Riverine density	0.127	0.064	NA	NA	−0.149	0.036	NA	NA	0.296	0.049	NA	NA	–	–	NA	NA
Wet tundra patch	−0.666	0.207	−0.561	0.109	−0.512	0.108	−0.538	0.079	−0.323	0.158	−0.391	0.100	−0.278	0.086	−0.313	0.070
Wet tundra density	–	–	NA	NA	−0.062	0.031	NA	NA	–	–	NA	NA	−0.039	0.024	NA	NA
Sedge-grass meadow density	0.337	0.072	NA	NA	0.272	0.038	NA	NA	0.213	0.051	NA	NA	0.363	0.029	NA	NA
Tussock tundra patch	−0.215	0.180	−0.116	0.104	0.085	0.086	0.027	0.057	−0.083	0.124	−0.036	0.083	0.019	0.065	0.061	0.050
Tussock tundra density	−1.684	0.124	NA	NA	−0.613	0.041	NA	NA	–	–	NA	NA	–	–	NA	NA
Moss-lichen patch	−0.977	0.235	−0.907	0.147	−0.768	0.133	−0.599	0.102	−0.143	0.225	−0.453	0.155	−0.272	0.121	−0.321	0.107
Moss-lichen density	−0.283	0.069	NA	NA	−0.111	0.037	NA	NA	0.095	0.050	NA	NA	–	–	NA	NA
Dwarf shrub patch	−0.112	0.600	−0.386	0.464	0.181	0.252	−0.127	0.187	−0.097	0.233	0.037	0.168	0.293	0.112	0.329	0.080
Dwarf shrub density	–	–	NA	NA	–	–	NA	NA	−0.301	0.070	NA	NA	−0.251	0.029	NA	NA
Low shrub patch	−0.467	0.641	−0.849	0.371	0.129	0.246	−0.076	0.183	−0.058	0.254	−0.005	0.203	0.500	0.133	0.424	0.106
Low shrub density	–	–	NA	NA	−0.193	0.040	NA	NA	–	–	NA	NA	0.176	0.028	NA	NA
Max-growth date	0.175	0.064	NA	NA	0.160	0.032	NA	NA	0.144	0.047	NA	NA	–	–	NA	NA
Distance to coastline	−0.789	0.064	−0.150	0.053	−0.331	0.039	–	–	−0.471	0.053	–	–	–	–	–	–
Precipitation	−0.230	0.086	NA	NA	0.385	0.041	NA	NA	−0.078	0.042	NA	NA	0.222	0.025	NA	NA
Elevation	–	–	–	–	–	–	–	–	–	–	−0.170	0.094	–	–	−0.166	0.066
Ruggedness	–	–	−0.059	0.040	–	–	0.031	0.022	–	–	0.051	0.039	–	–	0.079	0.021

1 Indicates variable was not included in initial set of variables considered for model.

2 Indicates variable was available to be included in model, but was not included in final model.

We divided each summer into five periods based on previously described life history traits for the herd ([11], adapted from Russell et al. [24] for the Porcupine Herd which also resides on Alaska’s north slope): calving (1–15 Jun), post-calving (16–30 Jun), mosquito (*Culex* spp.) harassment (1–15 Jul), oestrid fly (*Hypoderma* spp. and *Cephenemyia* spp.) harassment (although mosquitos may also still be present; 16 Jul –7 Aug), and late summer (8 Aug –15 Sep). While there is likely annual variation of when these periods occur, we feel these dates adequately capture the majority of each biological period annually and the relevant selective forces acting that we hoped to estimate with the RSFs. Additionally, we divided each of the calving, mosquito harassment, and oestrid fly harassment seasons’ data into two distinct groups. For calving, we partitioned the data into parturient (including calves that died; n = 21) and non-parturient females (n = 23). We divided mosquito and oestrid seasons into periods when mosquitoes or oestrid flies were predicted to be highly active and periods when they were not predicted to be highly active.

**Figure 3:**
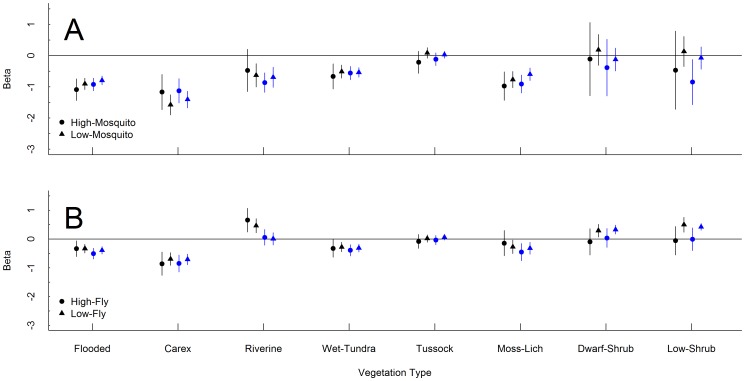
Coefficient estimates for vegetation patch types during insect periods. Results are from top resource selection models at the landscape (black) and patch (blue) scales during the (A) mosquito and (B) oestrid fly periods of summer for the Teshekpuk Caribou Herd in Alaska, 2004–2010. Coefficient estimates with 95% CI overlapping the zero-line are considered not significantly different than selection for sedge-grass meadows (used as the reference class in all resource selection function models).

We obtained an index of mosquito and oestrid fly activity based on hourly weather observations at Inigok (69 59.377 N 153 05.630 W) and Fish Creek weather stations (70 20.114 N 152 03.120 W; F. Urban, United States Geological Survey, unpublished data). Weather stations were located inland, southeast of Teshekpuk Lake in areas used by caribou during midsummer. Based on air temperature and wind speed we calculated mosquito and oestrid fly activity indices using the formulas of Russell et al. [24] derived from the northern Yukon Territory, Canada. Russell et al. [24] systemically sampled mosquitos and related their presence to wind speed and temperature to derive an index of activity. For mosquitos, the index identifies the highest probability of occurrence when wind is 0 m/s and temperature is >18°C. Conversely, the index identifies the lowest probability of occurrence when wind is >6 m/s and temperature is <6°C. Intermediate values were based on how far from the maximum temperature and minimum wind speed the weather observations were. Russell et al. [24] observed a strong relationship between the index and mosquito activity, with mosquitos being more frequent when the index was >0.5. Thus, if the index of mosquitoes being active was >0.5 for either weather station in the preceding 12 hours of a caribou location, we considered mosquitoes to be active. We did the same for determining oestrid fly activity, although the index derived by Russell et al. [24] for flies was based on a literature review and not field sampling. The highest probability of fly occurrence occurred when temperature was >18°C and wind was 0 m/s. The index of fly occurrence was lowest when temperature was <13°C and wind >9 m/s.

**Figure 4:**
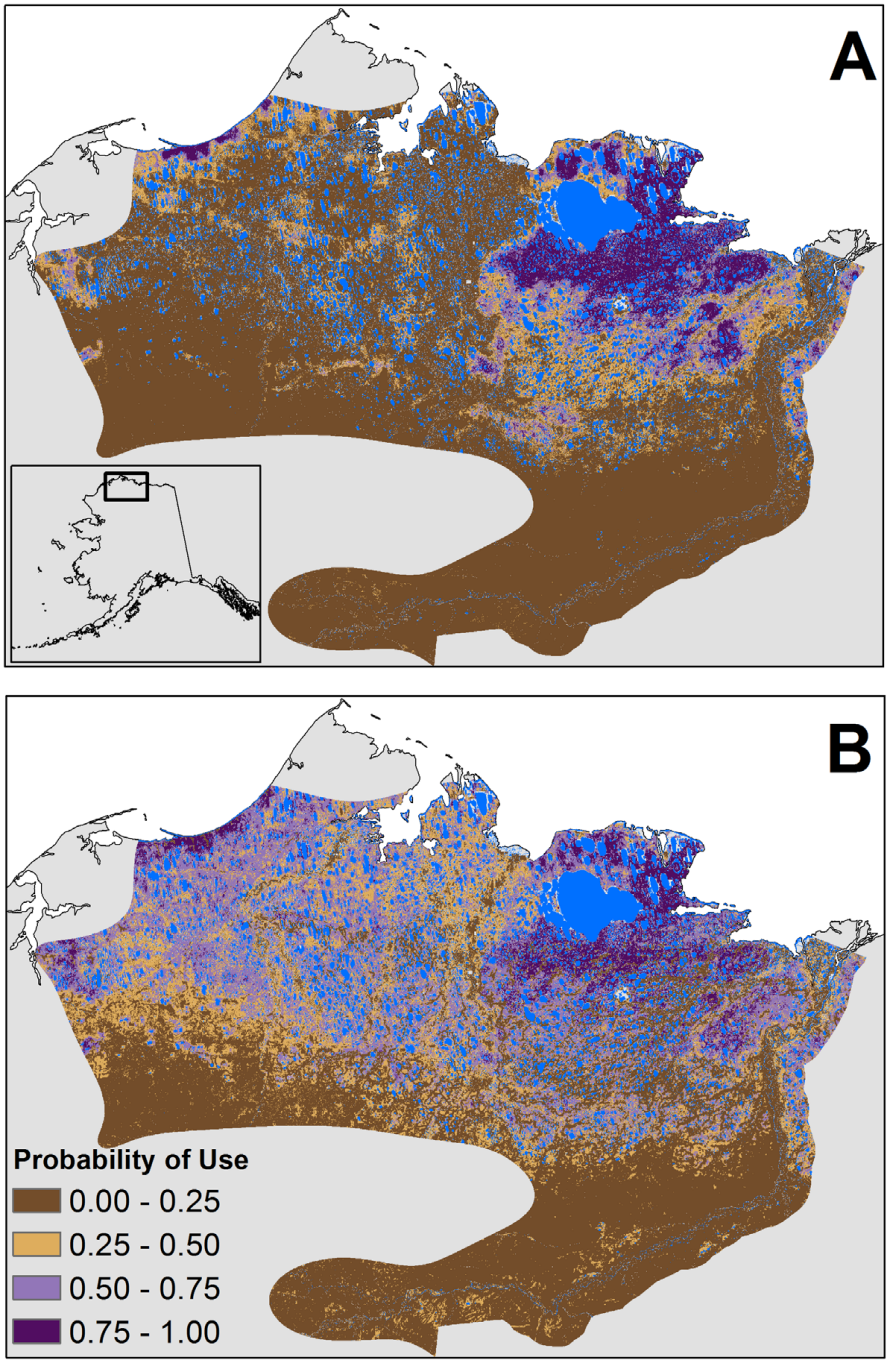
Relative probability of use for parturient and non-parturient females during calving. Map of relative probability of use during the calving period for (A) parturient and (B) non-parturient females of the Teshekpuk Caribou Herd derived from landscape-scale resource selection function results on data from 2004–2010. Blue pixels represent areas with water where resource selection was not measured. The predicted map’s extent is defined by the range of the herd during the calving period.

**Figure 5:**
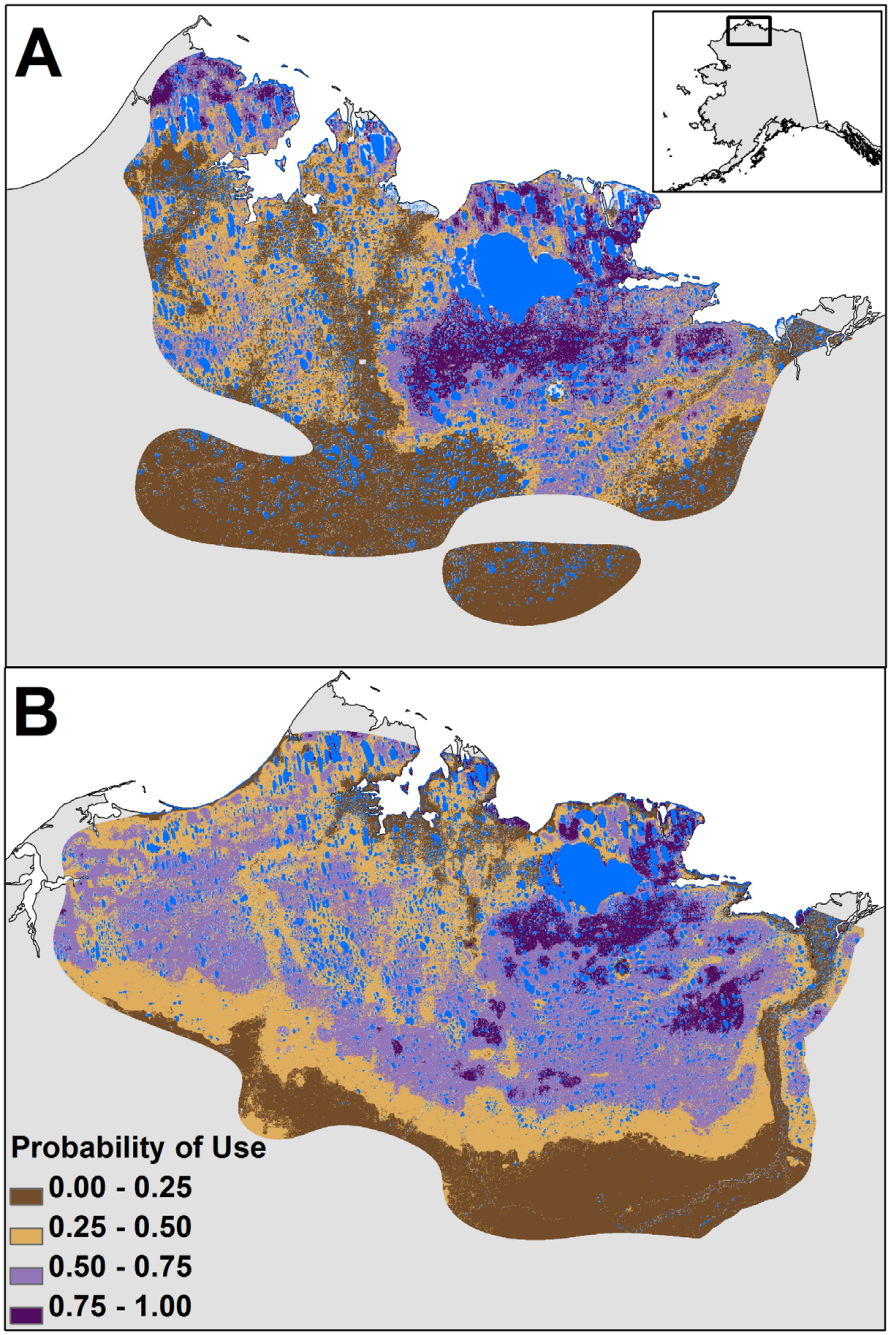
Relative probability of use during post calving and late summer. Predicted maps derived from landscape-scale resource selection function results for (A) post-calving and (B) late summer of the Teshekpuk Caribou Herd in Alaska with data from 2004–2010. Blue pixels represent areas with water where resource selection was not measured. The predicted maps’ extents are defined by the range of the herd during post-calving and late summer.

### Habitat Variables

Previous studies on resource selection by caribou in the Arctic have highlighted the importance of vegetation type and indices of quality at multiple scales [17], [20], weather [25], and topography [26] in describing patterns of selection. Thus, we included a suite of variables that represented these different categories. We used the vegetation classification provided by the Bureau of Land Management and Ducks Unlimited [27] to classify vegetation for the entire NPRA (pixel size of 30 m). The map was derived from Landscape Thematic Mapper satellite imagery and correctly classified 85% of general vegetation classes (e.g., shrub), and 75% of minor vegetation classes [27]. We collapsed the original 14 vegetation classes into 9 (Table 1). For all models, sedge-grass meadow was used as the reference class given the importance of plant species comprising this class to caribou in northern Alaska [17], [28]. We also determined the density of pixels (from the vegetation map described above) of each vegetation type across the study area following the methods of Johnson et al. [29]. Briefly, this method determines the scale (i.e., distance) at which variation in the distribution of a vegetation type is highest across the landscape by varying the size of window density is estimated over (i.e., three-term local quadrat variance; [30]). A moving window algorithm is then used to determine the density of the given vegetation type within a window having length and width equal to the scale of maximum variation identified above.

**Figure 6:**
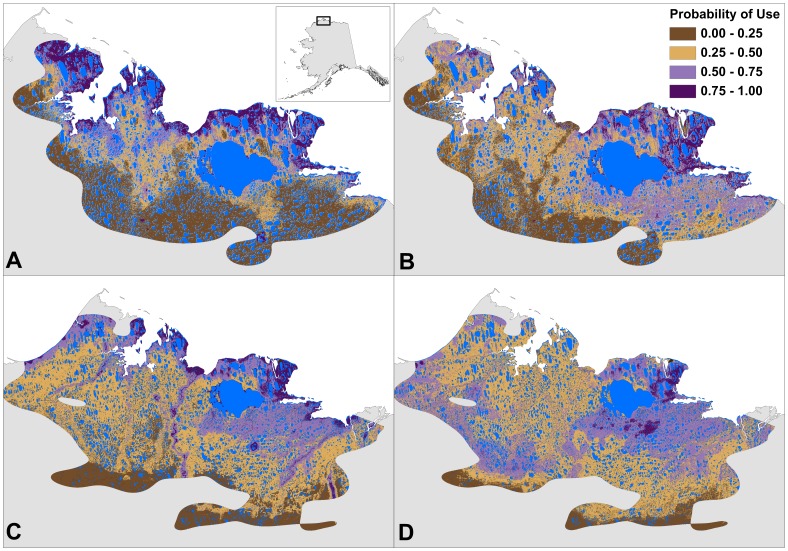
Relative probability of use during high and low insect harassment periods. Predicted maps derived from landscape-scale resource selection function results for the Teshekpuk Caribou Herd in Alaska based on data from 2004–2010: (A) high mosquito harassment, (B) low mosquito harassment, (C) high oestrid fly harassment, and (D) low oestrid fly harassment. Blue pixels represent areas with water where resource selection was not measured. The predicted map’s extent is defined by the range of the herd during the mosquito harassment period (A-B) and the oestrid fly harassment period (C-D).

To determine dates of green-up, maximum plant growth, and senescence across the study area each year, we used normalized difference vegetation index (NDVI) from SPOT satellite imagery (http://free.vgt.vito.be/home.php, accessed 9 Aug 2011). SPOT imagery is provided as 10 day composite images of NDVI and thus minimizes the potential for large areas to be masked by cloud cover. Conversely, it provides a lower temporal resolution of plant phenology, thus it is only serves as a general metric of phenology across the landscape. We defined date of green-up for each pixel as the day that the NDVI value exceeded background values of no-growth (i.e., >0.1, [31]). We defined date of senescence for each pixel as the day when NDVI dropped below background values of no-growth (i.e., <0.1). Finally, we defined date of max growth as the date when the maximum NDVI value was observed at each pixel (1 km). These metrics provide an index of forage biomass as there is a strong correlation between NDVI and green biomass for wet-sedge tundra in northern Alaska [32]. Additionally, these metrics provide a crude index of plant quality across the landscape as earlier plant growth is generally more digestible and higher in nitrogen in northern Alaska [33].

We used a 60 m Digital Elevation Model to obtain elevation data (United States Geological Survey National Elevation Dataset; http://ned.usgs.gov/, accessed 9 Aug 2011). We used the digital elevation model to calculate terrain ruggedness using the vector ruggedness measure with an 8 pixel window following the methods of Sappington et al. [34]. This method measures the dispersion of orthogonal vectors to the terrain’s surface [34]. We also determined the distance to the coastline across the study area. Finally, we obtained monthly PRISM precipitation data from the study area averaged across the years it was available (2000–2006; Scenarios Network for Alaska Planning, http://www.snap.uaf.edu/downloads/alaska-climate-datasets, accessed 9 Aug 2011). PRISM data had a resolution of 2 km.

We tested for colinearity between explanatory variables by calculating variance inflation factors (VIF) with the ‘corvif’ function found in the AED library in R [35]. We excluded a variable that had a VIF >3 [35], and then recalculated VIF for the remaining variables. We iterated this process until all variables had a VIF <3. The variables most often thrown out were elevation, dwarf shrub density, and tussock tundra density. For both scales of selection, we restricted our analysis to points where caribou were not sedentary (i.e., movement >25 m between relocations) in a first attempt to reduce autocorrelation in the data, reducing the data for analysis by 3%. However, we retained the first location in a bout of sedentary movement. Measurements of elevation and distance to coast were log transformed and terrain ruggedness square root transformed to meet normality assumptions. Additionally, we normalized each variable by subtracting each observed value from the variable’s mean and then dividing that value by the variable’s standard deviation to aid in model convergence [35]. When we detected non-linearities for elevation, distance to coast, or terrain ruggedness, we added a square term for that variable [36].

### Data Analysis

We estimated resource selection at two separate scales. At the landscape scale, we estimated selection within the herd’s range for a given period. We produced range maps for each period of summer in the study by estimating kernel density estimates [37] and defined the boundary as that area encompassed by the 95% isopleth using the ad hoc method to define the bandwidth parameter [38]. We generated one random location per observed location within the extent of the herd’s range during a given period (i.e., calving: n = 15,862; post-calving: n = 13,844; mosquito: n = 16,468; oestrid fly: n = 27,716; late summer: n = 52,824). We estimated landscape-scale RSFs with logistic generalized linear mixed models [39] using the ‘lmer’ function in the lme4 package in R [40]. We controlled for unequal sampling between years and individuals by estimating random intercepts for years and individuals.

To estimate patch-scale selection we used conditional logistic regression following the methods of Forester et al. [41]. For each used location, we generated five random locations by randomly selecting (with replacement) movement rates and bearings from the set of all movement rates and bearings between sequential locations for that caribou in that summer period. We used the ‘clogit’ function in the survival package for R [42] to estimate patch-scale RSFs, with each set of random and observed points assigned a unique cluster identity.

The resolution of explanatory variables varied such that many variables had grain sizes too coarse to be meaningful in the patch-scale RSFs. Thus, for these models we ran models with all possible combinations of vegetation type, elevation, distance to coast, and terrain ruggedness. We selected the best model in our set of candidate models based on the lowest Akaike’s Information Criterion (AIC) score [43]. We controlled for serial autocorrelation following the methods of Forester et al. [41]. This included calculating the sum of the deviance residuals for each strata (i.e., clusters or individuals for patch-scale and landscape scale RSFs, respectively) and fitting an intercept-only linear mixed effects model to the residuals grouped by strata. We then determined the correlation lag in the residuals and used this lag to split data into two independent groups. We reran the best model with these two datasets and calculated a robust covariance matrix from the average covariance matrix of the two data sets. The robust covariance matrix then provided adjusted standard error for estimates of model coefficients. For a more detailed explanation of the method see Forester et al. [41]. For both scales of analysis we considered regression coefficients to be significantly different than 0 if their 95% CI did not overlap 0. We also determined the predictive capacity for each model we used k-fold cross validation [44].

Finally, we created maps to visualize the results of each RSF by using the logistic regression equation to convert each model’s results into values ranging from 0 to 1 [8],
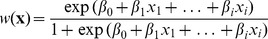
where *w*(**x**) is the relative probability of a pixel being selected, β_0_ is the estimated intercept, and β_i_ is the coefficient estimate for variable x_i_.

## Results

### Selection through Summer

All of the top RSF models had high k-fold cross-validation scores that indicated good predictability, except for the patch-scale model for late summer (Table 2). At both spatial scales, caribou consistently avoided patches of vegetation with high amounts of standing water (i.e., wet tundra, flooded, and *C*. *aquatilis*) throughout summer (Fig. 2A). Caribou also shifted selection for vegetation types as summer progressed and this pattern was generally consistent across both spatial scales (Fig. 2B). At the landscape scale, caribou consistently selected areas with high densities of sedge-grass meadows and *C*. *aquatilis*, but selection varied for other vegetation types through summer (Tables 3, 4, and 5). Selection for plant phenology at the landscape scale shifted from areas having later dates of plant growth initiation (i.e., green-up) during calving, to areas having later dates of maximum plant growth during the remaining periods of summer (Tables 3, 4, and 5). Additionally, caribou only selected areas on the landscape with more rugged terrain during the calving period (Table 4) but showed no preference the rest of the summer or at the patch-scale (Tables 3, 5).

### Parturient vs. Non-parturient

Selection of vegetation patches did not differ markedly between parturient and non-parturient females during calving at either spatial scale (Table 3). At both scales, parturient females generally avoided patches of most vegetation types compared to sedge-grass meadow (Table 3). The magnitude of selection, however, did vary between parturient and non-parturient females, but only at the landscape scale (Table 3). At the landscape scale, parturient females showed much stronger selection for areas with a high density of sedge-grass meadow and *C*. *aquatilis* (Table 3). Additionally, at the landscape scale, parturient females selected slightly more rugged terrain than non-parturient females (Table 3).

### High vs. Low Predicted Insect Harassment

Resource selection patterns differed between periods with high and low predicted insect harassment. At both spatial scales, caribou tended to select areas closer to the coastline when mosquito harassment was predicted to be high compared to when it was predicted to be low (Table 4). At both scales, caribou selected vegetation patches similarly between periods with high and low predicted mosquito harassment (Fig. 3A). At the landscape scale, however, when mosquito harassment was predicted to be high caribou showed stronger selection for areas with a high density of riverine vegetation but showed stronger avoidance of areas with a high density of tussock tundra compared to when harassment was predicted to be low (Table 4).

There were few differences in resource selection for vegetation patch type between periods with high and low predicted fly harassment (Fig. 3B). At the landscape scale, caribou selected areas with a higher density of riverine vegetation and closer to the coastline during periods of high harassment compared to periods of low harassment (Table 5). Additionally, caribou selected areas with a high density of low shrub when fly harassment was low, but showed no selection for those areas when harassment was high (Table 5).

### Predicted Probability of Use

Predicted maps of the relative probabilities of use, derived from the landscape-scale RSFs, showed a high concentration of preferred use areas surrounding Teshekpuk Lake during all periods of summer. This was especially true for the distribution of areas with a high probability of use for parturient females (Fig. 4A) and during post-calving (Fig. 5A). The distribution of areas with a high probability of use were more diffuse for non-parturient females during calving (Fig. 4B). Different patterns of predicted use areas were apparent between periods with high and low insect harassment (Fig. 6). During both mosquito and oestrid fly seasons, areas with high probabilities of use were adjacent to the coastline when harassment was high, but moved further inland when harassment was low. Additionally, when oestrid fly harassment was high, the relative probability of use was higher along river corridors (Fig. 6C) than during low fly harassment (Fig. 6D), or periods when mosquito harassment was high (Fig. 6A). During late summer, areas with a high probability of use were widely distributed across the herd’s range, but still showed a higher concentration of use surrounding Teshekpuk Lake (Fig. 5B).

## Discussion

Our results have implications for understanding the potential effects of oil and gas development on the TCH. Our landscape-scale RSF indicates that areas with characteristics currently selected by parturient caribou of the TCH are primarily concentrated in one contiguous area north, east, and south of Teshekpuk Lake. This suggests that if parturient females were displaced from the current calving ground, and selection patterns remain similar to those we estimated, they might be unable to find sufficient calving habitat having a similar composition within the historical distribution of the herd. The development of oil and gas infrastructure in the calving range of the Central Arctic Herd was followed by shifts in the distribution of concentrated calving areas for the western segment of the herd [4], [13], and they exhibited some evidence of reduced productivity [4]. Such impacts could have long-term negative population consequences for the TCH if alternative high value habitat is not available elsewhere. Thus, given the observed sensitivity of females with calves to development during the calving season, care must be taken to avoid developing a large proportion of these areas.

Our results also highlight important movement corridors between areas selected during periods with predicted low and high insect harassment. During periods with predicted low harassment, caribou selected areas further inland, presumably to take advantage of better foraging conditions. Once insect harassment was predicted to increase, caribou selected areas nearer the coastline where conditions would be less amenable for insect harassment. For caribou to move from habitat selected during low harassment to insect relief areas requires them to pass through two narrow corridors on the western and northeastern sides of Teshekpuk Lake previously identified by Yokel et al. [45]. Our results further highlight the importance of maintaining passage through these corridors. Additionally, because the primary areas for insect relief are concentrated along coastal margins and river drainages near Teshekpuk Lake, care must be taken to avoid developing these areas, given the importance of insect avoidance for caribou energetics [46]–[47].

Our use of RSFs for identifying areas of high value for the TCH allowed us to not only identify why the herd selected particular areas during summer, but also determine where else on the landscape such features occurred. This is a key difference from how caribou ranges are often characterized, such as using aerial surveys or telemetry data to look for areas with concentrated use. As Taillon et al. [7] observed, taking observational approaches can lead to significant mismatch in areas set aside for conservation, and those required by the herd. Resource selection functions can help quantify how much and where high value habitat exists on the landscape.

This is another important distinction between the methods we employed and the methods typically used to identify areas of concentrated use. Studies looking at development impacts on caribou often look at how far animals are displaced from development [4]–[5], but rarely quantify how much high value habitat was lost (but see [20]). By using RSFs to create spatial maps of predicted habitat value, managers can quantify how much high value habitat would be affected by different development plans and how much would be left if a particular area were developed, given documented patterns of avoidance by other herds (e.g., [4]). This will ultimately help find a better balance between allowing development to proceed while minimizing impacts to populations. Additionally, if managers chose to provide protection to areas identified as having a high probability of use based on RSFs, they can have greater certainty that they are capturing the range of natural variability in space use during a given period of the year.

In addition to providing information to help inform land-use decisions, our analysis helps increase our understanding of the herd’s summer ecology. Caribou exhibited a trend for greater selectivity of vegetation type for the period from post-calving through the period with oestrid flies, compared to late summer and calving when a wider variety of vegetation types were selected at similar levels to sedge-grass meadow at both scales. This is consistent with observations from other caribou herds in the arctic [48]–[50] and with changes in diet observed for the TCH during late summer [21].

Caribou selection for patches of different vegetation types generally did not vary between the two spatial scales. One explanation for this lack of pattern is the strong latitudinal gradient in vegetation types in northern Alaska leading to low environmental heterogeneity in the distribution of vegetation types. This same pattern of scale-invariant selection for vegetation types has been shown for muskoxen in the arctic (*Ovibos moschatus*) which forage on similar plant types as caribou during winter [51]. Caribou in the arctic display scale-dependent selection for resources but it is typically at the scale of individual plants [17], [28], [52]. At the two scales of selection we studied, we would not expect to see differences in selection for vegetation types between scales, but instead, changes in selection for their phenology. By using growth curves for individual plants and associated nutritional value at each phenological state, Finstad [53] was able to produce spatial maps of nutritional value for reindeer across the Seward Peninsula, Alaska through time which showed substantial spatial variation in nutritive value of reindeer forage. Detecting changes in individual plant quality was beyond the scope of our study, but would likely provide additional insight into how selection for vegetation changes at finer spatial scales.

The two scales of analysis were particularly informative during the calving period. Although parturient and non-parturient females did not show vastly different resource selection patterns at the patch-scale, their landscape-level resource selection differed. Parturient females showed substantially higher selection for areas with high densities of sedge-grass meadows than non-parturient females. Sedge-grass meadows at our study site are dominated by cotton grass (*Eriophorum* sp.) and *C*. *aquatilis* which have been shown to provide parturient caribou with highly digestible and high nitrogen content forage during lactation in northern Alaska [54]–[55]. Parturient females face significantly greater energetic costs than non-parturient females both before and after parturition [56]–[57]. Thus, selecting areas with higher densities of presumed high quality forage likely help parturient females meet these energetic demands.

The higher energetic requirements and intestinal changes of parturient females are predicted to lead to different space use patterns compared to non-parturient female ungulates [58]. As in other northern Alaska herds, parturient females in the TCH tend to arrive on calving grounds a week or two before non-parturient females to access newly emerged vegetation [59]. The relative immobility of neonatal calves [25] and the potential for reduced predation when calves are particularly vulnerable [17] may require parturient females to forgo short-term gains in quality and quantity found outside of the calving grounds in order to coincide the peak energetic demands of lactation with peak levels of forage quality. This was the result of Griffith et al [17] for the Porcupine Caribou Herd which found that at the landscape scale, parturient females selected calving grounds based on forage quality of the area rather than biomass. Our predicted maps of use by parturient females showed a marked difference in space use during the calving season compared to non-parturient females. While we did not detect differences in selection for plant phenology (as measured by NDVI) between parturient and non-parturient females, this may have been a result of the temporal scale of our phenology data. Parturient females, however, tended to select areas on the landscape with slightly rugged terrain which have been shown to lead to differences in the quality of forage available early in summer within the calving ground of the Central Arctic Herd [26]. Additionally, parturient females in the TCH have been observed further north in years of earlier snowmelt [25] which is consistent with females tracking snowmelt and newly emerged cotton grass flowers for their higher nutritional quality.

During periods with insects, our landscape-scale RSFs revealed that caribou selected areas on the landscape that have climatic conditions associated with lower rates of insect harassment such as near the coastline or along river terraces where wind and cooler temperatures reduce insect activity [21], [60]. Our landscape RSF results are similar to patterns of caribou space use observed by others when insect harassment was high (i.e., closer to the coast, riverine areas; [17], [60]–[61]. When selection was viewed at the patch-scale, however, there were few differences in selection between periods of high and low insect harassment. This might be because when harassment is low, caribou select areas on the landscape with a high density of preferred vegetation types (e.g., sedge-grass meadows), but when harassment increases, they shift to areas having lower levels of these vegetation types but with preferred climatic conditions. Once there, however, they prefer to occupy patches of the same vegetation type they did when harassment was low.

Similar to Witter et al. [62] for arctic caribou in Canada and Murphy and Lawhead [61] for the Central Arctic Herd, we observed different responses by caribou to a predicted increase in harassment by flies and mosquitoes. When the mosquito harassment was predicted to be high in our study, caribou tended to show greater selection for areas closer to the coastline than when oestrid fly harassment was predicted to be high. Additionally, caribou avoided riverine vegetation when predicted mosquito harassment was high, but selected those areas predicted high oestrid fly harassment. These differences are probably the result of both different behavioral responses for insect species, as was noted for the Bathurst Herd [62], and the TCH using areas further from the coast during late summer as indicted by Person et al. [11]. Caribou during the period of summer with oestrid flies would tend to be further from coastal relief areas and therefore might need to rely on other areas of the landscape providing relief from fly harassment such as gravel bars in rivers.

Our conclusions about the effects of insect activity on selection by the herd should be viewed cautiously as we relied on a coarse index of insect activity without any associated monitoring of how the index related to actual measures of insect abundance. When Russell et al. [24] created the index for the Porcupine Caribou Herd in northeastern Alaska, however, they related its value to mosquito abundance sampled throughout the summer and found good correspondence. A more recent study of the area surrounding Teshekpuk Lake [21], however, found that mosquitos had a lower threshold for wind (4 m/s compared to 6 m/s from Russell et al. [24]). Thus, our results are likely conservative, with some periods we predicted as having high mosquito activity actually being periods of low activity. Either way, we still observed significant differences in selection patterns and maps of predicted relative probability of use between periods with predicted high and low activity that match with results expected from previous studies of herds in northern Alaska [11], [45], [60].

Our study highlights the importance of maintaining existing calving areas for the herd because there are limited areas across the NPRA that share features the herd currently selects during calving and post-calving seasons. Additionally, given the importance of the coastline for mosquito relief, care must be taken to avoid hindering movement through the narrow corridors on either side of Teshekpuk Lake that much of the herd uses to reach the coastline [11], [45]. Our results also provide the baseline for use in a cumulative effects analysis of the potential impacts to TCH habitat from future oil and gas development across NPRA similar to Johnson et al. [20]. Our results can also help aid land managers in deciding which development plans will be least disruptive to caribou habitat (e.g., [63]). We additionally suggest that if pre-development spatial data exist for other herds, it would be informative to estimate the proportion of high-value habitat lost to development and what the demographic effects this had on herds. A retrospective analysis could help us to understand thresholds of habitat availability needed to maintain stable populations after development occurs. Finally, future research should address how use of preferred areas on the landscape relates to calf survival and summer weight gain. This would be beneficial for providing a more thorough understanding of the demographic effects of resource selection patterns observed in this study.
